# Clinical characteristics and statin eligibility of patients under 50 with ST‐elevation myocardial infarction

**DOI:** 10.1002/clc.24231

**Published:** 2024-02-16

**Authors:** Ayman Haq, Evan Walser‐Kuntz, Abdulrahman Gamam, Alexis Albers, Aaron Bae, Gretchen Benson, Michael D. Miedema

**Affiliations:** ^1^ Division of Cardiovascular Medicine Abbott Northwestern Hospital Minneapolis Minnesota USA; ^2^ Nolan Family Center for Cardiovascular Health Minneapolis Heart Institute Foundation Minneapolis Minnesota USA

**Keywords:** atherosclerotic cardiovascular disease, primary prevention, risk stratification, STEMI, tobacco, young adults

## Abstract

**Aims:**

This study seeks to understand the clinical characteristics, risk factors, and statin eligibility of younger adults who present with STEMI.

**Methods:**

We performed a retrospective analysis of a prospective cohort of STEMI patients <50 years. Baseline characteristics, medical history, prior medications, drug use, lipid profiles, cardiovascular risk factors were examined. Ten‐year ASCVD risk was calculated utilizing the Pooled Cohort Equations. Statin eligibility was determined according to the 2019 American College of Cardiology (ACC)/American Heart Association (AHA) and the 2022 US Preventive Services Task Force (USPSTF) guidelines.

**Results:**

Six hundred and thirty‐five individuals were included, the majority were men (82.4%) and white (89%), with a median age was 46.9 [42.0–48.0]. The most prevalent risk factors were current smoking (59%), hyperlipidemia (44%), and hypertension (37%). Drug use was rare (8.3%). Preventative medication use was low, aspirin was the most common (14%), followed by ACE inhibitors/ARBs (12%), statins (11%), and beta‐blockers (9.1%). Mean HDL‐C was low at 36.4 ± 12.0 mg/dL, while mean LDL was unremarkable at 112.4 ± 37.9 mg/dL. According to the 2019 ACC/AHA guidelines, 45.5% were classified as statin recommended, 8.7% were classified as statin considered, and 45.8% were classified as statin not recommended. According to the 2022 USPSTF guidelines, 29% were classified as statin recommended, 12.4% were classified as statin considered, and 58.6% were classified as statin not recommended.

**Conclusions:**

Younger adults with STEMI exhibit high rates of tobacco use and low rates of preventative medications use. Approximately half of the cohort did not meet criteria for statin initiation.

## Practitioner Points

- This is a cohort of patients <50 years who presented with ST‐elevation myocardial infarction, examining the baseline characteristics, medical history, prior medications, drug use, lipid profiles, cardiovascular risk factors and whether the patient's qualified for statin initiation before their presentation.
- Six hundred and thirty‐five individuals were included, the majority were men (82.4%) and white (89%), with a median age was 46.9 [42.0–48.0]. The most prevalent risk factors were current smoking (59%), hyperlipidemia (44%), and hypertension (37%). Drug use was rare (8.3%). Preventative medication use was low, aspirin was the most common (14%), followed by angiotensin converting enzyme inhibitors/angiotensin receptor blockers (12%), statins (11%), and beta‐blockers (9.1%). Mean HDL‐C was low at 36.4 ± 12.0 mg/dL, while mean LDL was unremarkable at 112.4 ± 37.9 mg/dL.
- According to the 2019 American College of Cardiology (ACC)/American Heart Association guidelines, 45.5% were classified as statin recommended, 8.7% were classified as statin considered, and 45.8% were classified as statin not recommended. According to 2022 US Preventive Services Task Force guidelines, 29% were classified as statin recommended, 12.4% were classified as statin considered, and 58.6% were classified as statin not recommended.

## Graphical abstract

Statin eligibility of STEMI patients under 50 according to the 2019 American College of Cardiology/American Heart Association (ACC/AHA) and the 2022 US Preventive Services Task Force (USPSTF) statin guidelines. 45.8% and 58.6% of individuals were classified as statin not recommended according to the 2019 ACC/AHA and 2022 USPSTF guidelines, respectively.

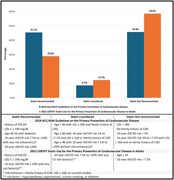

## 1 INTRODUCTION

Advances in cardiovascular prevention have led to a decrease in hospitalizations for acute myocardial infarction (MI) in the US over the past few decades.^1^, ^2^ However, recent data suggest that hospitalizations for acute MI in younger adults have not seen the same decline and may be increasing.^3^, ^4^, ^5^ One reason for this may be the earlier development of obesity and its associated cardiovascular risk factors. Multiple studies have demonstrated the increasing prevalence of obesity in adolescents and younger adults,^6^, ^7^ which is strongly associated with continued obesity in adulthood.^8^ This leads to earlier development of cardiometabolic risk factors including hyperlipidemia, hypertriglyceridemia, hypertension, and elevated blood glucose.^9^ Chronic exposure to these risk factors at earlier ages increases the risk of developing premature atherosclerotic cardiovascular disease (ASCVD). Younger individuals are less likely to undergo cardiovascular risk assessment and are less likely to be eligible for preventative therapies when they do undergo assessment.^10^ Ultimately, this allows the development of clinical ASCVD that may otherwise be attenuated.^11^

ST‐elevation myocardial infarction (STEMI) is a unique subset of MI associated with a high risk for morbid cardiovascular complications and therefore warrants emergent treatment regardless of age. Prevention of STEMI remains of utmost importance to the maintenance of optimal cardiovascular health. To optimize the approach to prevention of STEMI in younger adults, a better understanding of the demographics and risk factors in this population is needed. In this analysis, we sought to determine the clinical characteristics of young STEMI patients (<50 years), their cardiovascular risk at the time of STEMI, and their pre‐STEMI eligibilty for lipid‐lowering therapy utilizing data from a large contemporary cohort of patients treated in a regional STEMI system.

## 2 METHODS

### 2.1 Study design and patients

The Minneapolis Heart Institute (MHI) regional STEMI program includes Abbott Northwestern Hospital in Minneapolis, MN as well as over 20 regional hospitals.^12^ It was instituted in 2003, standardizing the timely treatment of patients with STEMI who present to any of the included hospitals, regardless of percutaneous coronary intervention (PCI) capabilities. Inclusion criteria were new ST‐elevation or new left bundle‐branch block on electrocardiogram with chest pain <24 h in duration. There were no exclusion criteria, including age. Patient data are collected in a prospective and standardized database for quality improvement and clinical research purposes. Follow‐up data is collected for a period of 5 years following the index admission. We examined all patients aged 50 or younger from this database from March 2003 to June 2021. Clinical documentation and the initial catheterization report for each individual was reviewed and those that did not meet the above STEMI criteria were excluded. For patients who had multiple admissions meeting criteria for STEMI, the first admission was considered the index admission for this analysis. This study was funded entirely by the Minneapolis Heart Institute Research Foundation. Institutional review board approval was obtained before data collection and data analysis. No data were collected on patients who had not consented to allow their clinical data to be used for research purposes.

### 2.2 Data collection

Patient baseline factors including age, sex, race, height, weight, systolic blood pressure, and hemoglobin a1c% were collected from the electronic health record (EHR). Body mass index (BMI) was calculated from the individual's weight in kilograms and height in meters. Hemoglobin A1c% values within 6 months of the patient's index admission were included in this study. Histories of coronary artery disease (CAD), MI, PCI, coronary artery bypass graft surgery (CABG), stroke, hypertension, hyperlipidemia, diabetes, and family history of CAD were collected from the EHR and confirmed by the admitting physician during the admission interview. Home medications were collected from the EHR and were verified with either the patient or with the patient's outpatient pharmacy by a registered nurse. If this was not possible, the patient's primary care physician was contacted to verify the patient's home medications. A history of drug use was ascertained by the admitting physician during the admission interview and confirmed by reviewing prior urine drug screen results from the EHR. The decision to check a urine drug screen during the index admission was determined by the treating physician. Total cholesterol, LDL‐C, HDL‐C, and triglycerides drawn within 24 h after the index admission were collected from the EHR.

### 2.3 Statistical analysis

Baseline characteristics were stratified by sex when possible and compared using Wilcoxon rank‐sum tests, Fisher's exact tests, and Pearson's *χ* ^2^ tests as appropriate. Categorical variables were presented as frequencies and percentages and continuous variables were summarized as median and interquartile range (IQR).

The 10‐year ASCVD risk was calculated by the Pooled Cohort Equations (PCE).^13^ Due to incomplete antihypertensive medication data and the fact that the only systolic blood pressure available was taken during hospitalization for STEMI, the values for systolic blood pressures for all patients were imputed via the following criteria: (1) 130 mmHg—no history of hypertension, (2) 135 mmHg—a history of hypertension and also on a beta‐blocker, angiotensin‐converting‐enzyme inhibitor, or angiotensin receptor blocker, (3) 145 mmHg—a history of hypertension and not on any of the aforementioned medications. Data were also imputed for missing lipid profile values. A sensitivity analysis was performed without imputation which excluded individuals with missing data, as their ASCVD risk could not be calculated. All statistical analyses were performed with RStudio version 4.2.3.^14^ Multiple imputation using the *mice* package in R was used to deal with missingness in the PCE variables.^15^

### 2.4 Statin eligibility classification

Individuals were classified into three groups, statin recommended, statin considered, or statin not recommended according to the 2019 American College of Cardiology (ACC)/American Heart Association (AHA) guidelines on the primary prevention of cardiovascular disease and the 2022 US Preventive Services Task Force (USPSTF) guidelines on statin use for the primary prevention of cardiovascular disease in adults.^13^, ^16^ This classification incorporated both the 10‐year ASCVD risk according to the PCE, as well as clinical factors including a history of ASCVD, diabetes, smoking, hyperlipidemia, hypertension, family history of CAD, and LDL‐C levels.

## 3 RESULTS

A total of 821 patients met inclusion criteria for this sample. Of these, 186 patients (23%) had a nonatherosclerotic etiology of their STEMI, leaving 635 patients with STEMI due to atherosclerotic disease available for analysis. The majority of the cohort were men (82.4%) and identified as white (89%) (Table 1). The median age was 46.0 [42.0–48.0] years, with a heavily left‐skewed distribution of 524 patients (83%) age 41–50 years, while only 8 (1.3%) were age 21–30 years. The median BMI was 29.0 [26.0–33.0] kg/m^2^ in men and slightly higher in women (31.0 [27.2–36.0] kg/m^2^). Systolic blood pressure on admission was similar in both genders. The most prevalent risk factors were current smoking (59%), hyperlipidemia (44%), and hypertension (37%). Nearly three‐quarters of the cohort were either current or former smokers (74%). A family history of CAD was also prevalent in over half of the cohort (52%). In comparison, diabetes was not as prevalent (13%), with a median hemoglobin A1c of 5.80 [5.40–6.80] %, although it was more prevalent in women (22%) than in men (11%) (*p* = .001). A small proportion had a history of CAD (11%), while a history of stroke was rare (1.3%); rates did not differ between genders.

**Table 1:** Baseline characteristics, medical history, home medications, and drug use on admission.

	Overall, *N* = 635^a^	Female, *N* = 112^a^	Male, *N* = 523^a^	*p* Value^b^
*Baseline characteristics and medical history* c				
Age	46.0 (42.0, 48.0)	46.0 (42.0, 48.0)	46.0 (42.0, 48.0)	0.8
21–30	8 (1.3%)	0 (0%)	8 (1.5%)	
31–40	103 (16%)	16 (14%)	87 (17%)	
41–50	524 (83%)	96 (86%)	428 (82%)	
Race				0.062
White	568 (89%)	96 (86%)	472 (90%)	
Black	29 (4.6%)	11 (9.8%)	18 (3.4%)	
Asian	15 (2.4%)	1 (0.9%)	14 (2.7%)	
Latino	10 (1.6%)	2 (1.8%)	8 (1.5%)	
Other	8 (1.3%)	2 (1.8%)	6 (1.1%)	
Unknown	5 (0.8%)	0 (0%)	5 (1.0%)	
BMI	29.5 (26.0, 34.0)	31.0 (27.2, 36.0)	29.0 (26.0, 33.0)	0.002
A1c%	5.8 (5.4, 6.8)	5.9 (5.4, 7.2)	5.8 (5.4, 6.7)	0.5
Smoking				0.4
Current	372 (59%)	72 (64%)	300 (57%)	
Former	93 (15%)	14 (12%)	79 (15%)	
Never	170 (27%)	26 (23%)	144 (28%)	
Diabetes	82 (13%)	25 (22%)	57 (11%)	0.001
Hypertension	233 (37%)	42 (38%)	191 (37%)	0.8
Hyperlipidemia	281 (44%)	47 (42%)	234 (45%)	0.6
CAD	72 (11%)	12 (11%)	60 figur(11%)	0.8
PCI	49 (7.7%)	7 (6.2%)	42 (8.0%)	0.5
MI	49 (7.7%)	9 (8.0%)	40 (7.6%)	0.9
CABG	4 (0.6%)	2 (1.8%)	2 (0.4%)	0.15
CVA	8 (1.3%)	2 (1.8%)	6 (1.2%)	0.6
Family history of CAD	326 (52%)	55 (50%)	271 (52%)	0.7
*Home medications* d				
Aspirin	90 (18.7%)	17 (19.5%)	73 (18.6%)	0.8
P2Y12i	12 (1.9%)	1 (0.9%)	11 (2.1%)	0.7
Statin	73 (15.2%)	10 (11.5%)	63 (16.0%)	0.5
ACEi/ARB	78 (13.9%)	12 (12.4%)	66 (14.2%)	0.6
Beta blocker	58 (10.3%)	15 (15.3%)	43 (9.3%)	0.2
*Drug use*				
Cocaine	13 (2.0%)	4 (3.6%)	9 (1.7%)	0.3
Methamphetamine	13 (2.0%)	3 (2.7%)	10 (1.9%)	0.7
Marijuana	27 (4.3%)	3 (2.7%)	24 (4.6%)	0.4
*Predicted 10‐year ASCVD risk by pooled cohorts equation*				
Mean (SD)	0.07 (0.05)	0.05 (0.04)	0.07 (0.05)	<0.001
Median (IQR)	0.06 (0.03, 0.09)	0.04 (0.02, 0.06)	0.06 (0.03, 0.09)	<0.001

^a^ Median (IQR); *n* (%).

^b^ Wilcoxon rank sum test; Fisher's exact test; Pearson's *χ* ^2^ test.

^c^ Individuals with missing data were excluded from this analysis: hemoglobin a1c% (367), BMI (3).

^d^ Individuals with missing data were excluded from this analysis: aspirin (155), P2Y12i (2), statin (155), ACEi/ARB (75), beta blocker (74).

Preventative medication use was low throughout the cohort. Aspirin was the most common medication (14%), followed by angiotensin‐converting enzyme inhibitors/angiotensin receptor blockers (12%), statins (11%) and beta‐blockers (9.1%). Rates did not differ between genders. Drug use in this cohort was uncommon, although not insignificantly so. In total, 53 patients (8.3%) had a history of marijuana (4.3%), methamphetamine (2.0%), or cocaine use (2.0%). The median 10‐year ASCVD risk was low, although slightly higher in women compared with men (6 [3–10] % vs. 4 [2–6] %, *p* < .001) (Table 1).

Mean HDL‐C was low in the cohort at 36.4 ± 12.0 mg/dL, while mean LDL was relatively unremarkable at 112.4 ± 37.9 mg/dL (Table 2). Mean LDL was higher in men than in women (115.0 ± 38.7 mg/dL vs. 99.7 ± 30.9 mg/dL, *p* < .001). Mean triglycerides were borderline high at 181.5 ± 149.8 mg/dL.

**Table 2:** Lipid profile by gender.

	Overall, *N* = 635^a^	Female, *N* = 112^a^	Male, *N* = 523^a^	*p* Value^b^
Total cholesterol	183.6 (43.7)	171.9 (36.6)	186.0 (44.7)	0.001
HDL‐C	36.4 (12.0)	38.5 (13.5)	35.9 (11.7)	0.086
LDL‐C	112.4 (37.9)	99.7 (30.9)	115.0 (38.7)	<0.001
Triglycerides	181.5 (149.8)	175.7 (159.9)	182.7 (147.8)	0.075

*Note*: Individuals with missing data were excluded from this analysis: total cholesterol (98), HDL‐C (100), LDL‐C (115), triglycerides (97).

^a^ Mean (SD).

^b^ Wilcoxon rank sum test.

Lipid profiles did not significantly differ between patients who were taking a statin before admission and those who were not (Table 3). Mean LDL was somewhat lower in men on a statin compared with men who were not a statin (97.7 ± 45.2 mg/dL vs. 118.6 ± 36.4 mg/dL, *p* < .001), but this relationship was not found in women who were and were not on a statin (100.3 ± 20.0 mg/dL vs. 100 ± 32.5 mg/dL, *p* = .922). Mean HDL did not differ based on statin use in men (35.2 ± 8.0 mg/dL vs. 35.4 ± 10.1 mg/dL, *p* = .974) or in women (38.0 ± 10.9 mg/dL vs. 38.3 ± 14.4 mg/dL, *p* = .988). This was also observed in mean triglycerides in men (164.5 ± 92.7 mg/dL vs. 184.3 ± 140.3 mg/dL, *p* = .323) and in women (198.1 ± 158.1 mg/dL vs. 176.3 ± 169.7 mg/dL, *p* = .976) (Table 3).

**Table 3:** Lipid profile by gender and statin use.

	Men			Women		
	No, *N* = 330^a^	Yes, *N* = 63^a^	*p* Value^b^	No, *N* = 77^a^	Yes, *N* = 10^a^	*p* Value^b^
Total cholesterol	189.0 (43.9)	167.3 (51.3)	<0.001	172.8 (37.9)	168.9 (30.9)	0.655
HDL‐C	35.4 (10.1)	35.2 (8.0)	0.974	38.3 (14.4)	38.0 (10.9)	0.988
LDL‐C	118.6 (36.4)	97.7 (45.2)	<0.001	100.0 (32.5)	100.3 (20.0)	0.922
Triglycerides	184.3 (140.3)	164.5 (92.7)	0.323	176.3 (169.7)	198.1 (158.1)	0.976

*Note*: Individuals with missing data were excluded from this analysis: lipid profile/statin use (155).

^a^ Mean (SD).

^b^ Wilcoxon rank sum test.

**Figure 1:**
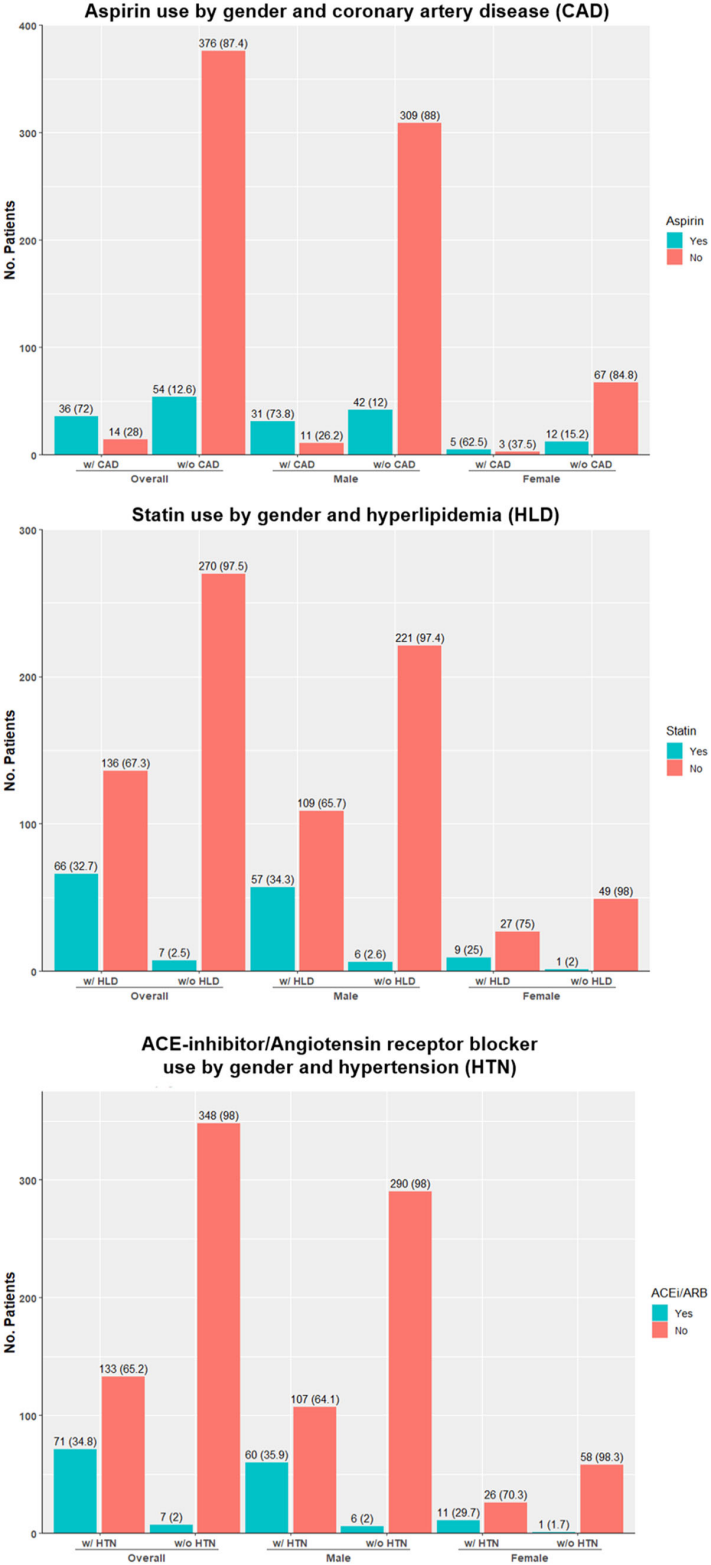
Preventative medication use by relevant risk factor and gender. Individuals with missing data were excluded from this analysis: aspirin (155), statin (156), ACEi/ARB (76).

Rates of preventative medication use were higher when stratified by the relevant risk factor. Overall, 72% of individuals with a history of CAD were taking aspirin before admission. However, only 32.7% of individuals with a history of hyperlipidemia were on a statin and 34.8% of individuals with a history of hypertension were on an ACEi/ARB. Rates of aspirin, statin, and ACEi/ARB use were lower in individuals without the relevant risk factor (12.6%, 32.7%, and 2.0% respectively) (Figure 1).

**Figure 2:**
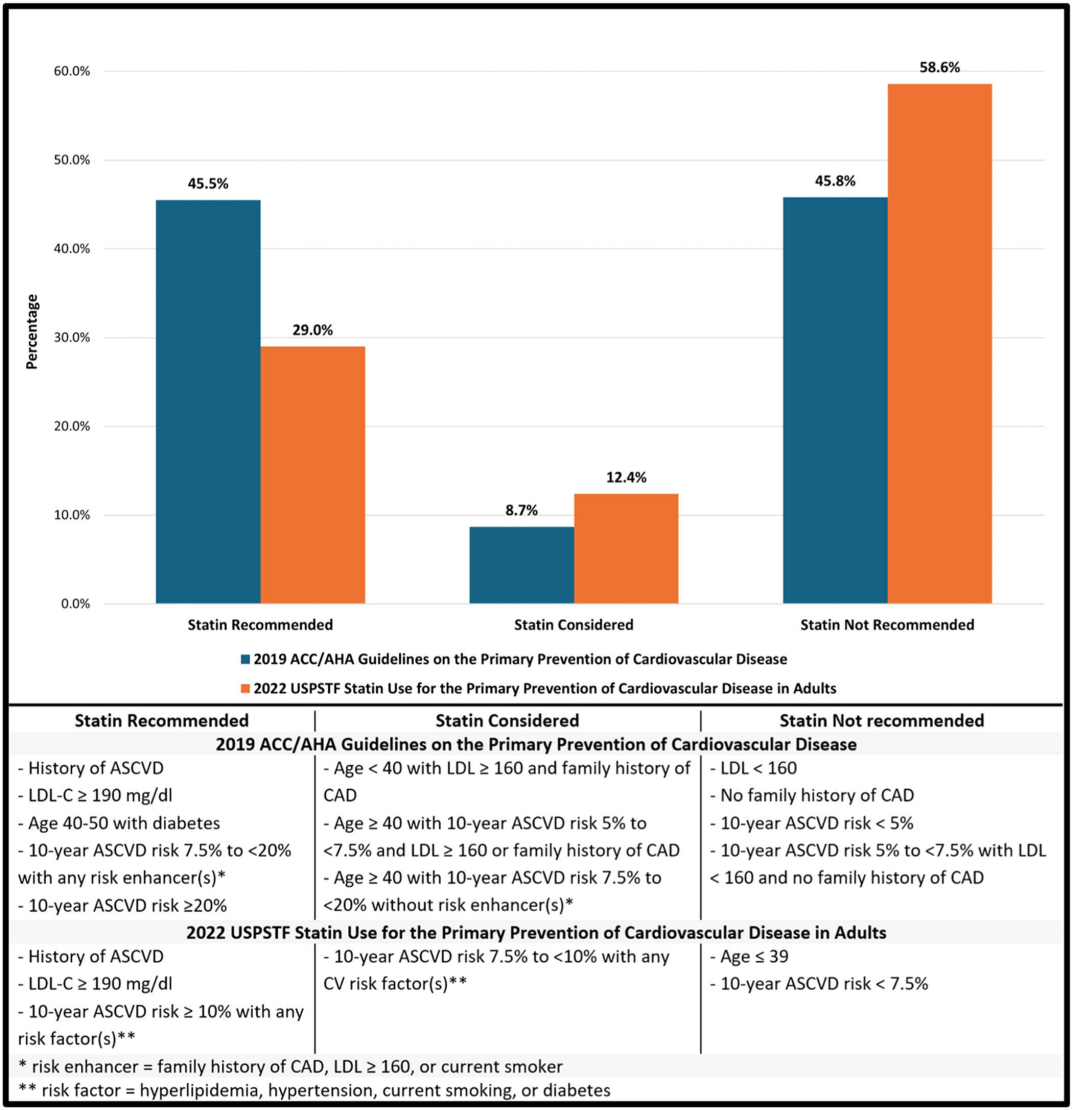
Statin eligibility of STEMI patients under 50 according to the 2019 American College of Cardiology/American Heart Association and the 2022 US Preventive Services Task Force statin guidelines.

According to the 2019 ACC/AHA guidelines for the primary prevention of cardiovascular disease, 45.5% (289) of individuals were classified as statin recommended and 8.7% (55) of individuals were classified as statin considered. 45.8% (291) of individuals were classified as statin not recommended. According to the 2022 USPSTF guidelines, only 29% (184) of individuals were classified as statin recommended, 12.4% (79) were classified as statin considered, and 58.6% (372) of individuals were classified as statin not recommended (Figure 2). The sensitivity analysis included 505 individuals, with a similar percentages assigned to each statin recommendation group for both the 2019 ACC/AHA guidelines and 2022 USPSTF guidelines analyses (Supporting Information: Table 1).

## 4 DISCUSSION

We performed a retrospective analysis of a prospective cohort of 635 patients aged ≤50 years who presented with STEMI due atherosclerotic disease to examine the clinical characteristics, risk factors, medication use, and statin eligibility based on the most recent guidelines for this population. The most prevalent risk factor was smoking, followed by hyperlipidemia and hypertension. Diabetes and drug use were relatively uncommon. Median LDL was not significantly elevated in the cohort and approximately half of young individuals experiencing STEMI were not eligible for statin therapy before their event.

### 4.1 Comparison to YOUNG‐MI registry

Prior cohorts have examined MI in younger adults. The Partners YOUNG‐MI registry retrospectively examined a cohort of 2097 patients aged ≤50 years who presented with MI.^17^ While this cohort included patients with NSTEMI and patients with nonatherosclerotic coronary disease (spontaneous coronary artery dissection, MI with nonobstructive coronary arteries), many of its findings mirror our analysis. Most of the patients were men at 80.7%. Hyperlipidemia, smoking, and hypertension were the most prevalent risk factors at 91.1%, 50.8%, and 47.8%, respectively. Only 19.8% of the cohort had diabetes at the time of their admission and rates of drug use (cocaine or marijuana) was also low at 10.9%. Lipid panels were remarkably similar as well. The mean total cholesterol was 192 mg/dL, with mildly depressed HDL‐C of 37 mg/dL, a mildly elevated LDL‐C of 119 mg/dL, and normal median triglyceride of 150 mg/dL. The median predicted 10‐year ASCVD risk score was slightly lower at 3.9%–5.3%, although approximately 5.7% of those who underwent coronary angiography did not have any atherosclerotic disease.

### 4.2 Comparison to 2017–2020 NHANES

While cardiovascular risk factors are prevalent throughout the cohort, examining them in light of the prevalence of risk factors in the general adult population in the United States yields additional insights. When compared to data in adults ≥20 years from the 2017–2020 National Health and Nutrition Examination Survey, the prevalence of hypertension was somewhat lower in this cohort (36.7% vs. 46.7%) while the prevalence of hyperlipidemia was somewhat higher (44.2% vs. 34.7%).^18^ Diabetes was similarly prevalent in both groups (12.9% vs. 14.1%). Total cholesterol in this cohort was similar to that in adults ≥20 years (189.0 vs. 187.2 mg/dL), however, HDL‐C was lower (35.4 vs. 53.6 mg/dL) and triglycerides were higher (148 vs. 91.6 mg/dL). HDL‐C was similar in both current smokers and never smokers in our cohort (36.0 vs. 37.2 mg/dL, *p* value = .325), suggesting that the lower HDL‐C is not secondary to cigarette smoking.^19^ Further, only 11 individuals had an LDL‐C ≥ 190 mg/dL, suggesting that familial hyperlipidemia was not significantly prevalent in our cohort.

In contrast, the rates of current smoking were substantially higher in this cohort than in adults ≥20 years. According to data from the 2020 National Health Interview Survey, only 12.5% (95% CI, 11.9%–13.0%) of adults ≥18 years reported cigarette use every day or some days, compared with 58.6% of this cohort, representing a four to fivefold higher rates of smoking compared to the general adult population.^20^

### 4.3 AMIYA and BMC2 studies

Further evidence that smoking may play a crucial role in STEMI secondary to atherosclerotic coronary disease in younger adults comes from an interesting cohort from India.^21^ Notably, only patients aged ≤30 years with STEMI were included in the study. In total, it included 1116 patients over a 2‐year period with a mean age of 26.3 years. Smoking was the most prevalent risk factor in the cohort at 78.5%. The next most prevalent was a family history of premature CAD at 46.8%, followed by hyperlipidemia at 21.2% hypertension at 20.5%, and diabetes at 17.2%. On coronary angiography, 80.6% had obstructive atherosclerotic CAD, while another 12.2% had nonobstructive atherosclerotic CAD. Only 5.2% had normal coronary arteries and 2% had spontaneous coronary artery dissection.

An analysis of the Blue Cross Blue Shield of Michigan Cardiovascular Consortium registry revealed similar findings. A total of 6892 individuals who underwent primary PCI for STEMI were included. Overall, the rate of smoking in the entire cohort was 46.43%. In individuals aged 18–34, the rate of smoking was 78.02% compared with just 23.72% in the general population aged 18–34. The odds ratio for smoking for individuals in the registry was 3.4 (95% CI 3.3–3.4), while the odds ratio for smoking for individuals aged 18–34 was 11.4 (95% CI, 10.0–12.8), when compared to the general population matched for age.^22^

### 4.4 Preventative medication use

The use of preventive medications before MI in our cohort was low. These rates appear to be similar when compared to prior cohorts. In a Norwegian cohort of individuals aged <45, conducted from 2013 to 2016, only 12% of individuals were on aspirin, 13% were on a statin, 10% were on a beta blocker, and 11% were on an ACEi/ARB.^23^ When stratified by the relevant risk factor, rates of preventative medication use were substantially better. For example, most individuals with a history of CAD were on aspirin before admission, while a small minority of those without a history of coronary heart disease were on aspirin (Figure 1). This trend was also observed in individuals with hyperlipidemia and hypertension. Overall, statin use has been increasing over time.^24^, ^25^, ^26^ This may be in part due to the 2013 ACC/AHA guidelines expanding statin eligibility by recommending the use of the PCE, rather than just LDL‐C levels to determine statin eligibility.^27^, ^28^ This expansion has been shown to appropriately identify at risk individuals but has also been shown to overestimate ASCVD risk in certain populations.^29^, ^30^, ^31^, ^32^, ^33^ However, relative rates of statin utilization remain low. An analysis by Rochat et al. examined statin use during 2003–2006 and 2014–2017 in the prospective population‐based CoLaus|PsyCoLaus cohort.^26^ During the initial survey period, 29.6% of individuals were statin eligible, but only 24.0% of those eligible were taking a statin. Ten years later, 45% of the cohort was eligible for statin treatment, but a similarly small proportion of those eligible, 26.3%, were taking a statin. Moreover, only 15.8% and 28.2% of individuals taking a statin met LDL‐C treatment targets during the 2003–2006 and 2014–2017 surveys, respectively. Our data suggest that rates of preventative medication use in younger adults remains inappropriately low, in part due to the underestimation of ASCVD risk, and is part of a larger trend of the underutilization of preventative therapies in at‐risk populations.

### 4.5 Statin eligibility

Nearly half of the individuals in the present analysis were classified as statin not recommended according to the 2019 ACC/AHA 2019 guidelines.^13^ The most common reason for this classification was a 10‐year ASCVD risk <5% (29%), followed by age <40 without risk enhancers (11%), and a 10‐year ASCVD risk of 5%–7.5% without risk enhancers (6.5%). Over half of the individuals in the present analysis were classified as statin not recommended according to the 2022 USPSTF guidelines for either a 10‐year ASCVD risk <7.5% (47.1%) or age <40 (11.5%).^16^ The ACC/AHA 2019 guidelines were better able to classify high‐risk individuals by implementing a lower 10‐year ASCVD risk threshold to consider statin initiation (5% vs. 7.5%), a lower LDL‐C threshold to consider statin initiation (160 vs. 190 mg/dL) and by incorporating an early family history of CAD as a risk enhancer.

Both guidelines rely on the PCE to calculate baseline 10‐year ASCVD risk, which is likely poorly calibrated for younger individuals, driving the underclassification of ASCVD risk in this cohort. The Partners Young‐MI registry examined statin eligibility according to the 2013 ACC/AHA and the 2016 USPSTF guidelines with similar findings with 52% and 71% of the cohort classified as statin not recommended.^34^

### 4.6 Role of imaging in risk stratification

The benefit of noninvasive imaging for ASCVD risk stratification in younger adults is unclear. Coronary artery calcium scoring can be used to quantify calcified coronary plaques, however, younger individuals are more likely to have noncalcified plaque, even when presenting with acute coronary syndrome.^35^, ^36^, ^37^ A secondary analysis of the Prospective Multicenter Imaging Study for Evaluation of Chest Pain trial examined the use of coronary computed tomography angiography (CTA) in risk stratification.^38^ It included 3986 patients aged 60.5 ± 8.2 years and calculated statin eligibility by incorporating coronary CTA findings into the PCE risk model. In statin naïve patients, this approach did improve the overall discrimination of the risk model. Event rates in statin eligible patients increased from 3.4% to 4.0% and decreased in statin non‐eligible patients from 2.7% to 2.1%, when incorporating coronary CTA findings. However, this was largely due to the appropriate downward reclassification of approximately 17% the cohort from statin eligible to statin not‐eligible. Hence, coronary CTA may not provide the incremental risk stratification to appropriately reclassify younger adults as higher risk for ASCVD.

An alternative clinical model for assessing longer‐term ASCVD risk in younger adults was proposed by Pencina et al.^39^ They followed 4506 individuals aged 20–59 without prior ASCVD, from the Framingham Offspring cohort. Male gender, age, smoking, systolic blood pressure, antihypertensive treatment, diabetes, total cholesterol positively correlated with a 30‐year ASCVD risk while HDL‐C had a negative correlation. These correlations remained significant when adjusting for changes in risk factors over time. Building on this, Stone et al. proposed adopting a 30‐year risk screening protocol in adults aged 20–39 years.^40^ If smoking, hypertension, family history of premature ASCVD, severe hyperlipidemia or familial hyperlipidemia, diabetes, or other persistent major enhancing risk factors are present, they proposed pursuing intensive lifestyle and pharmacological interventions as appropriate.

## 5 LIMITATIONS

While data were collected in a systematized, prospective manner, it was not collected exclusively for the purposes of clinical research and was analyzed retrospectively for the present manuscript. Data on common antihypertensive medication classes, including thiazides, calcium channel blocker, nitrates, and hydralazine was unavailable for this analysis, which prevents the classification of individuals on these medications into the appropriate hypertensive categories. To ameliorate this, we categorized any individual with a history of hypertension and not on any antihypertensive medications into the highest risk hypertensive category. Regardless, the missing medication data may affected the analysis in a manner that the authors were unable to predict. Most of the present cohort was White, which may limit the generalizability of our findings to other races/ethnicities. For example, a large retrospective analysis examined rates of statin eligibility and prescriptions across racial, ethnic, and language groups.^27^ After adjusting for gender, age, income status, insurance status, hypertension, hyperlipidemia, obesity, primary care utilization, and Medicaid expansion status Asian, Black, Latino and non‐English speaking White patients were more likely to be statin eligible than English speaking White patients. Of these, all but English‐speaking Black and Latino patients were more likely to be prescribed statins than English‐speaking White patients. It has been suggested that the lipid profile during an acute MI may underrepresent ASCVD risk.^41^ However, multiple prior sensitivity analyses have shown that the decrease in ASCVD risk is typically not clinically meaningful.^34^, ^42^ Data on a family history of premature CAD was unavailable. For this, we substituted any family history of CAD to allow for an overclassification of statin eligibility and a more conservative analysis. Baseline renal function data was unavailable for this analysis. This precluded the use of chronic kidney disease as an ASCVD risk‐enhancer when assessing for statin eligibility. However, the prevalence of chronic kidney disease in a sample of patients <50 years old is likely low and therefore unlikely to meaningfully alter the analysis.^43^ We were unable to account for less common risk enhancing factors, such as chronic inflammatory disease, HIV infection, a history of pre‐eclampsia or preterm delivery, persistently elevated inflammatory markers, abnormal ankle‐brachial index which may have led to the undercategorization of statin eligibility in individuals with these risk factors, according to the 2019 ACC/AHA guidelines. Additionally, several variables had high rates of missing data. Namely, over half of individuals had missing hemoglobin A1c% values. However, the median hemoglobin A1c% is consistent with the low rates of diabetes observed in this study. Similarly, lipid profile, aspirin, and statin data were unavailable in roughly 15%–24% of individuals in this study. We were able to adjust for this via imputation in the statin eligibility analysis. A sensitivity analysis excluding imputed data did not reveal substantial differences in statin eligibility.

## 6 CONCLUSION

Younger adults with STEMI exhibit high rates of tobacco use and low rates diabetes and drug use. Preventative medication use was also low in this cohort, which may in part be due to the underascertainment of ASCVD risk in this population using conventional risk assessment methods. Developing risk stratification methods better calibrated for a younger population may help identify and treat younger adults at high risk for an ASCVD event.

## AUTHOR CONTRIBUTIONS

*Design of the work*: Ayman Haq, Gretchen Benson, and Michael D. Miedema. *Data collection*: Ayman Haq, Abdulrahman Gamam, Alexis Albers, Aaron Bae, Gretchen Benson, and Michael D. Miedema. *Data analysis and interpretation*: Ayman Haq, Evan Walser‐Kuntz, Gretchen Benson, and Michael D. Miedema. *Drafting the article*: Ayman Haq, Evan Walser‐Kuntz, and Michael D. Miedema. *Critical revision of the article*: Ayman Haq, Evan Walser‐Kuntz, Abdulrahman Gamam, Alexis Albers, Aaron Bae, Gretchen Benson, and Michael D. Miedema. *Final approval of article*: Ayman Haq, Evan Walser‐Kuntz, Abdulrahman Gamam, Alexis Albers, Aaron Bae, Gretchen Benson, and Michael D. Miedema.

## CONFLICT OF INTEREST STATEMENT

The authors declare no conflict of interest.

## Supporting information

Supporting information.

## Data Availability

Research data are not shared.
