# Supplementary material for: Clinical characteristics and statin eligibility of patients under 50 with ST‐elevation myocardial infarction
Source: Clin Cardiol. 2024 Feb 16;47(2):e24231. doi: 10.1002/clc.24231 (PMC10870333; doi:10.1002/clc.24231)
Supplement: Supplementary file 1 — Supporting information. [file CLC-47-e24231-s001.docx]

| Supplementary Table 1: Sensitivity analysis for the 2019 ACC/AHA and 2022 USPSTF statin guidelines, without imputed data (N = 505) | | |
| --- | --- | --- |
| Statin Recommended, N (%) | Statin Considered, N (%) | Statin Not recommended, N (%) |
| 2019 ACC/AHA Guidelines on the Primary Prevention of Cardiovascular Disease | | |
| 223 (44.1%) | 42 (8.3%) | 240 (47.6%) |
| 2022 USPSTF Statin Use for the Primary Prevention of Cardiovascular Disease in Adults | | |
| 136 (26.9%) | 64 (12.7%) | 305 (60.4%) |
